# Chem2Bio2RDF: a semantic framework for linking and data mining chemogenomic and systems chemical biology data

**DOI:** 10.1186/1471-2105-11-255

**Published:** 2010-05-17

**Authors:** Bin Chen, Xiao Dong, Dazhi Jiao, Huijun Wang, Qian Zhu, Ying Ding, David J Wild

**Affiliations:** 1 School of Informatics and Computing, Indiana University, Bloomington, IN, USA; 2 School of Library and Information Science, Indiana University, Bloomington, IN, USA

## Abstract

**Background:**

Recently there has been an explosion of new data sources about genes, proteins, genetic variations, chemical compounds, diseases and drugs. Integration of these data sources and the identification of patterns that go across them is of critical interest. Initiatives such as Bio2RDF and LODD have tackled the problem of linking biological data and drug data respectively using RDF. Thus far, the inclusion of chemogenomic and systems chemical biology information that crosses the domains of chemistry and biology has been very limited

**Results:**

We have created a single repository called Chem2Bio2RDF by aggregating data from multiple chemogenomics repositories that is cross-linked into Bio2RDF and LODD. We have also created a linked-path generation tool to facilitate SPARQL query generation, and have created extended SPARQL functions to address specific chemical/biological search needs. We demonstrate the utility of Chem2Bio2RDF in investigating polypharmacology, identification of potential multiple pathway inhibitors, and the association of pathways with adverse drug reactions.

**Conclusions:**

We have created a new semantic systems chemical biology resource, and have demonstrated its potential usefulness in specific examples of polypharmacology, multiple pathway inhibition and adverse drug reaction - pathway mapping. We have also demonstrated the usefulness of extending SPARQL with cheminformatics and bioinformatics functionality.

## Background

Recent advances in chemical & biological sciences have lead to an explosion of new data sources about genes, proteins, genetic variations, chemical compounds, diseases and drugs. Through integrated and intelligent data mining, this information could provide important insights into the complex functions of biological systems and the actions of chemical compounds or drugs on these systems. However, this can only be achieved when data is semantically integrated (i.e. using multiple data sources that are connected in meaningful ways) and in particular when chemical and biological resources are brought together in such a framework [1,2].

There are critical problems in biology that can only be answered through computational analysis of this kind of integrated chemical and biological information. For example, it is considered increasingly important to profile existing and potential new drugs for their effects across many protein targets, not just a single target of interest (this is known as polypharmacology [3,4]). Only by exploring the relationships of the drugs to a wide body of target information can we determine this profile. Further, the polypharmacologic action of drugs on targets that fall within the same pathway can determine the drug's ability to interrupt pathways at multiple points, and thus provide more robust efficacy. Relationships between these pathways and potential side effects of drugs or chemicals that are being considered as drugs (such as undesirably inhibition of a pathway) can only be determined by large-scale analysis of the impact of the chemicals on known pathway systems [5,6]. The need to address these kinds of problems has led to the emergence of the field of *Systems Chemical Biology *[7], a field which covers the computational analysis of integrated chemical and biological information for the enhancement of biological understanding, including *chemogenomics *(the relationship of compounds to genes specifically).

Implementing such an integrated system involves the creation of large networks of linked compounds, protein targets, genes, pathways, drugs, diseases and side effects from multiple, heterogeneous sources. It must be possible to query these data in ways that go beyond querying of a single source and allow inferences that cross domains: for example a positive experimental test of a chemical compound in a biological enzymatic assay where the enzyme is associated with a particular metabolic pathway constitutes a probable action of that compound on the pathway. Currently, there are significant barriers to carry out this kind of analysis. Many of the needed data sources overlap and cover similar data (we refer to them as homogenous or semi-homogenous data sources) but with slightly different foci. All data sources tend also to be published in very diverse formats (text files, scholarly journal articles, XML, relational databases, and so on) and may be structured or unstructured. The semantic relationship of these datasets to each other is often unclear.

Recent Semantic Web technologies provide efficient ways to integrate heterogeneous data. The Semantic Web [8] initially proposed by Tim Berners-Lee, has demonstrated its utility in the life sciences, healthcare and drug discovery [2,9-11]. Various semantic languages have been established to represent and query semantic meaning of data and relationship. In this work we use RDF [12] to model chemogenomic and systems chemical biology data and use SPARQL [13] to query them.

A variety of RDF-based Semantic Web resources have already been created for biological data and drug data separately. *Bio2RDF *[14] provides a platform and a strategy for generation and querying of biological RDF data in a distributed framework, with around 4 billion RDF triples across over 30 biological resources. *Linking Open Drug Data *(LODD) [15] led by the W3C Semantic Web Health Care and Life Sciences Interest Group (HCLS IG) links RDF data from the Linked Clinical Trials dataset derived from ClinicialTrial.gov, DrugBank (a repository of almost 5000 FDA-approved drugs), and many other sources, with more than 8.4 million RDF triples and 388,000 links to external data sources. Similar efforts are YeastHub [16], LinkHub [17], BioDash [18] and BioGateway [19].

Approaches to querying across heterogeneous data sources in the life sciences have been discussed previously [20]. In the work reported in this paper, we have created an RDF resource for integrated chemical and biological information. We have further employed methods to facilitate the easy generation of SPARQL queries and have implemented a variety of searching options for the RDF datasets by extending the SPARQL query language to include domain-specific cheminformatics and bioinformatics functionality. We refer to this combination of new RDF triples, links to Bio2RDF and LODD, and searching capabilities as *Chem2Bio2RDF*. We present three specific examples of how Chem2Bio2RDF can be used in the previously described important areas of polypharmacology, pathway inhibition and adverse drug reaction analysis.

## Methods

### Datasets

Our datasets are organized into six categories based on the kinds of biological and chemical concepts they contain. These categories are: chemical & drug, protein & gene, chemogenomics, systems (i.e., PPI and pathway), phenotype (i.e., disease and side effect) and literature. Some data sources are listed in multiple categories. Some of the data used were previously employed in relational database format in our prior work [3] and in this case they were simply converted into RDF/XML via a D2R server [21]. For the rest of the datasets, we acquired the raw dataset (by downloading from web sites), and converted the data into our relational database using customized scripts. These are then published as RDF through the D2R server. The data can be queried via a D2R SPARQL endpoint.

**Figure 1:**
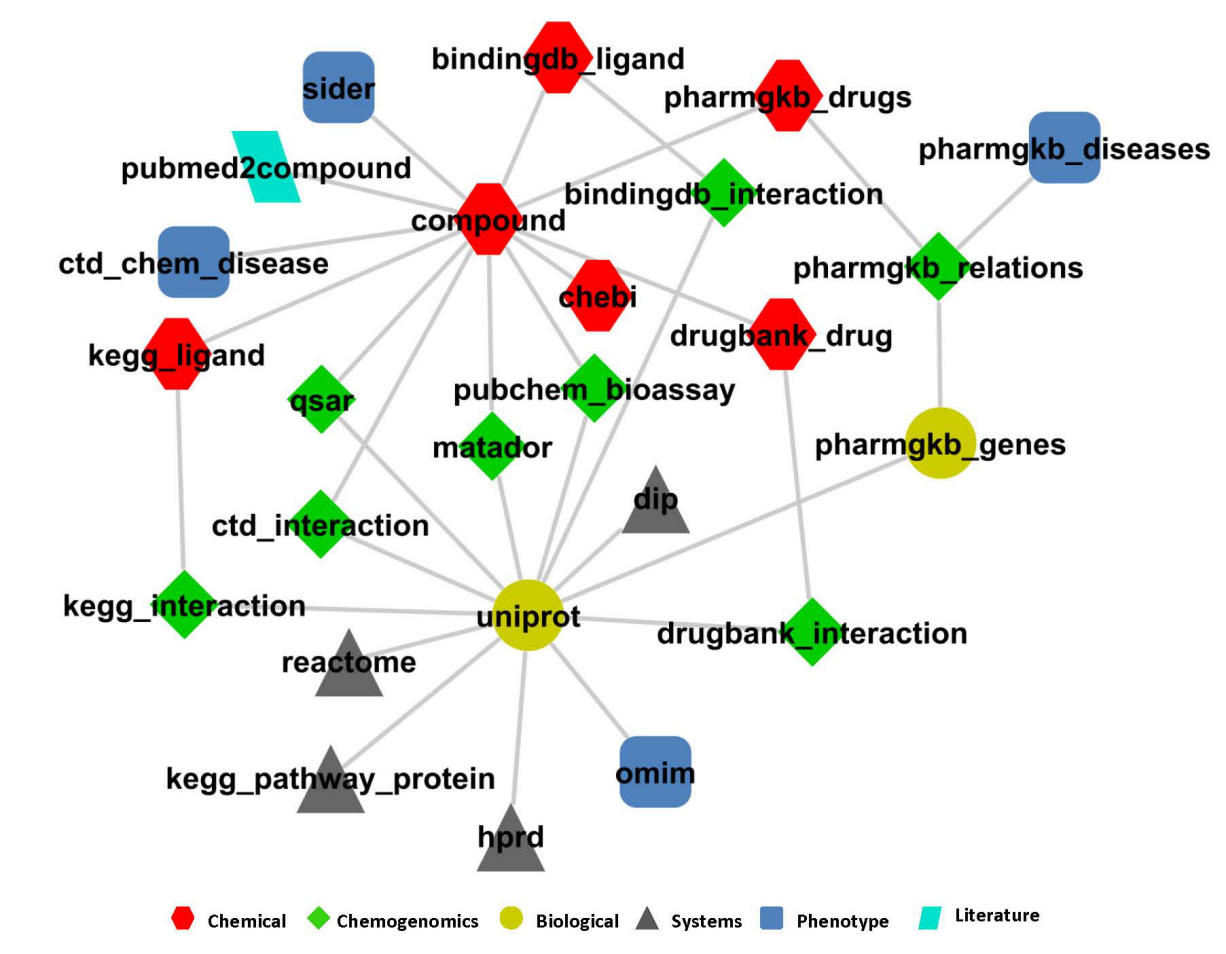
**Chem2Bio2RDF datasets**. Nodes represent data sources. Two nodes are linked if the data of one source is directed to the data of another source. The node is shaped and colored by its type, which is organized into six categories. Some databases map to multiple sources.

We adopted PubChem Compound ID (CID) as the identifier for compounds, and UniProt ID for protein targets. The compounds represented by other data formats (e.g., SMILES, InChi and SDF) were mapped to CID via InChi keys. A detailed description of each of the datasets can be found at http://chem2bio2rdf.org/datasets.html. All the triples are stored together and the whole set is called Chem2Bio2RDF dataset (Figure 1).

### Storage and querying architecture

**Figure 2:**
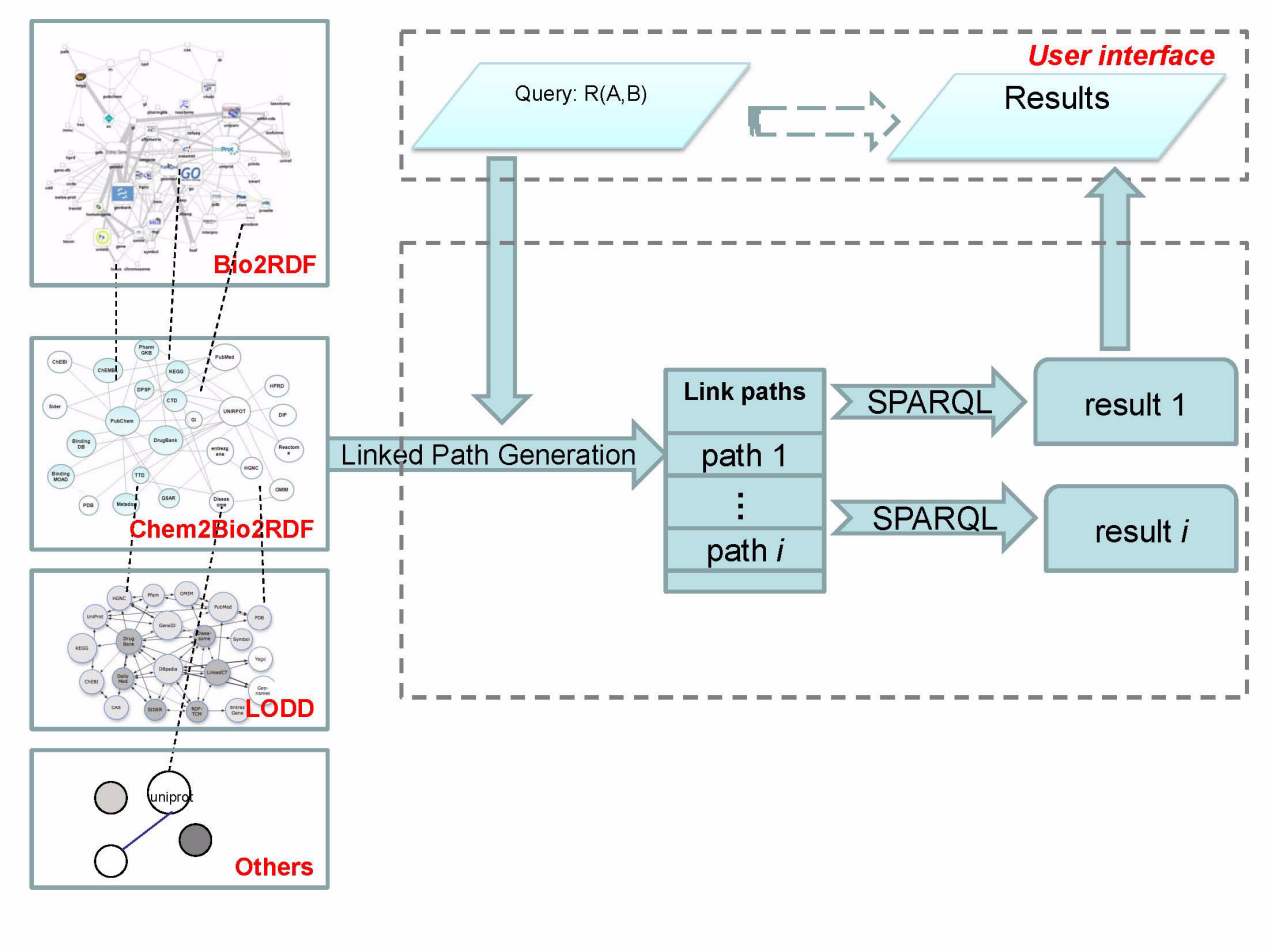
**Chem2Bio2RDF querying architecture**. Chem2Bio2RDF is linked to Bio2RDF, LODD and other RDF resources. LPG refers to prototype methods used for automatically generating links between two given objects and automated generation of SPARQL queries.

We developed a schema to classify the concepts and the RDF resources in Chem2Bio2RDF. The schema file can be downloaded from http://chem2bio2rdf.org/download.html. The RDF data can be explored and queried in our website http://chem2bio2rdf.org/. Figure 2 shows the generalized query architecture of Chem2Bio2RDF and how it links with the other sources (including Bio2RDF and LODD).

We linked our data to LODD and Bio2RDF using the owl:sameAs construct. Since LODD and BioRDF have strict namespace definition and dereferenceable URIs, it is straightforward to link them simply via a D2R mapping file. For example, the drug Lepirudin http://chem2bio2rdf.org/drugbank/resource/drugbank_drug/DB00001 is linked to the following URIs: http://bio2rdf.org/drugbank_drugs:DB00001, http://www.dbpedia.org/resource/Lepirudin, http://www4.wiwiss.fu-berlin.de/dailymed/resource/ingredient/Lepirudin, and http://www4.wiwiss.fu-berlin.de/drugbank/resource/drugs/DB00001

### Implementation of cheminformatics and bioinformatics functionality in SPARQL

SPARQL is a query language for RDF and provides functions and syntax to satisfy generalized querying needs. However, these basic functions are not able to address specific chemical/biological search needs. We extended SPARQL using the ARQ [22] in Jena with cheminformatics functionality from the Chemistry Development Kit (CDK) [23], ChemBioGrid [24], and bioinformatics functionality from BioJava [25]. We can now thus perform a diverse set of functions in a query including chemical similarity searching, protein similarity searching, and drug-like compound selection. For the chemical similarity search, we add two extending functions: *fingerprint *and *tanimoto *mapping to the CDK functions *getFingerprint *and *Tanimoto.calculate*. The fingerprint function generates a string of 166 binary descriptors that represent the presence (denoted as 1) or absence (denoted as 0) of common 2D structural features in a chemical as defined by the popular MACCS structural keys [26]. The Tanimoto Coefficient is used to calculate the similarity between these pairs of descriptors [27]. The Tanimoto coefficient between two chemicals *A *and *B *is defined as:

Where *N*_*C *_is the number of bits that are set in the fingerprints of both *A *and *B*, and *N*_*A *_*and N*_*B *_are the total number of bits set in A and B, respectively.

## Results

### Creation of the Chem2Bio2RDF repository

**Figure 3:**
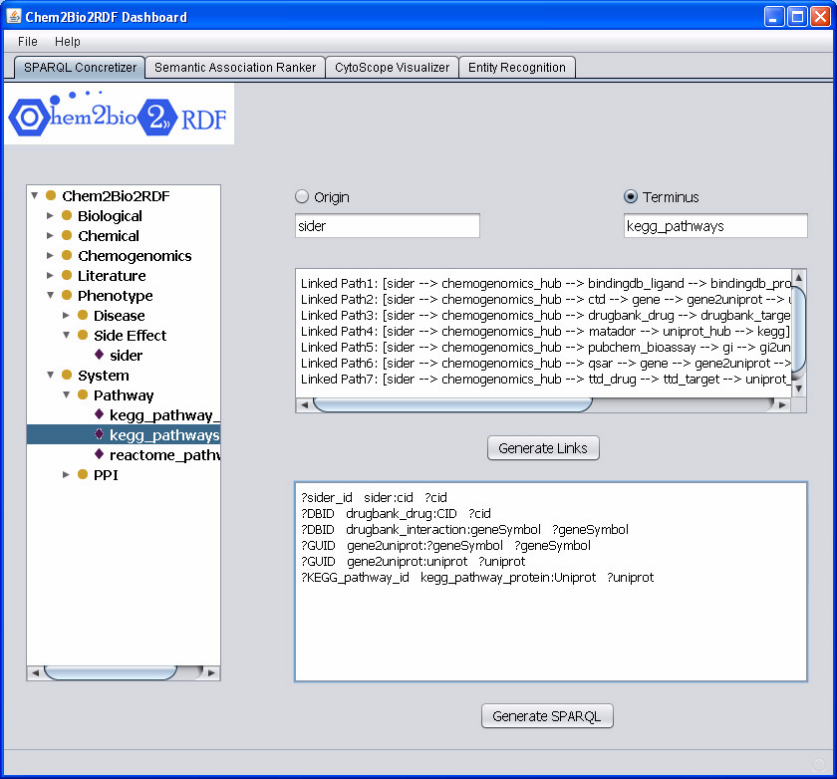
**Prototype linked path generation**. A prototype of a tool that allows users to select origin and terminal data sources. The tool will generate all the possible paths between the two data sources, will allow the user to select individual paths, and will then convert these into SPARQL queries.

We have created a single repository called Chem2Bio2RDF by aggregating data from multiple repositories including PubChem Bioassay [28], DrugBank [29], KEGG Ligand [30], CTD [31], BindingDB [32], PharmGKB [33], MATADOR [34], and a number of small QSAR sets available on the web [35]. A schema of the data sources has been created, and the data in these sets are represented as RDF triples, that link chemical compounds (as identified by a PubChem ID) with targets, genes, side effects, diseases and publications (Figure 1). Table 1 lists the datasets along with the number of triples in Chem2Bio2RDF. We have created a variety of prototype tools for querying the data, including one that allows automated generation of links between dataset resources (Figure 3)

**Table 1:** Chem2Bio2RDF datasets: some data sources map to multiple RDF resources.

Database	Resource name	Number of RDF triples
PubChem Compound	compound	233852

PubChem BioAssay	pubchem_bioassay	1715247

ChEBI	chebi	2237330

KEGG	kegg_ligand	96000

KEGG	kegg_interaction	70029

KEGG	kegg_pathway_protein	84760

CTD	ctd_interaction	2443826

CTD	ctd_chem_disease	2025513

BindingDB	bindingdb_ligand	223818

BindingDB	bindingdb_interaction	800016

PharmGKB	pharmgkb_drugs	14760

PharmGKB	Pharmgkb_genes	340808

PharmGKB	pharmgkb_relations	73276

PharmGKB	pharmgkb_diseases	9591

DrugBank	drugbank_drug	47640

DrugBank	drugbank_interaction	111001

Public QSAR sets	qsar	27269

MATADOR	matador	253488

UNIPROT	uniprot	34951

HPRD	hprd	408177

Reactome	reactome	21985

DIP	dip	1113840

OMIM	omim	23432

SIDER	sider	305510

PubMed	pubmed2compound	269178

### Case study 1: Linking DrugBank and PubChem to investigate Dexamethasone polypharmacology

Since approximately 35% of known drugs have more than one target, the efficacy of many drugs is increasingly thought to come from their effect on multiple targets. This is known as *polypharmacology*. We recently studied the utility of data in PubChem for identifying cases of polypharmacology [3] as well as how chemical and biological data can be mined on a large scale [36]. We can now extend this, using Chem2Bio2RDF, to incorporate data from DrugBank as well as PubChem. In particular, if a compound has the same multiple targets as a marketed drug but has a different chemical structure, that compound could be a candidate for a novel new therapy. Conversely, if we have already established polypharmacologic action of known drugs, can we find other interesting drug-like compounds that also show similar polypharmacology? These questions can be formulated as a query: *find all the drug-like compounds in PubChem BioAssay that share at least two targets with a drug in DrugBank*. We can now translate this into a SPARQL query of Chem2Bio2RDF (in this example using Dexamethasone - an anti-inflammatory 9-fluoro-glucocorticoid which interacts with six proteins - as the drug of interest). The exact SPARQL query used is available on the chem2bio2rdf.org website.

**Figure 4:**
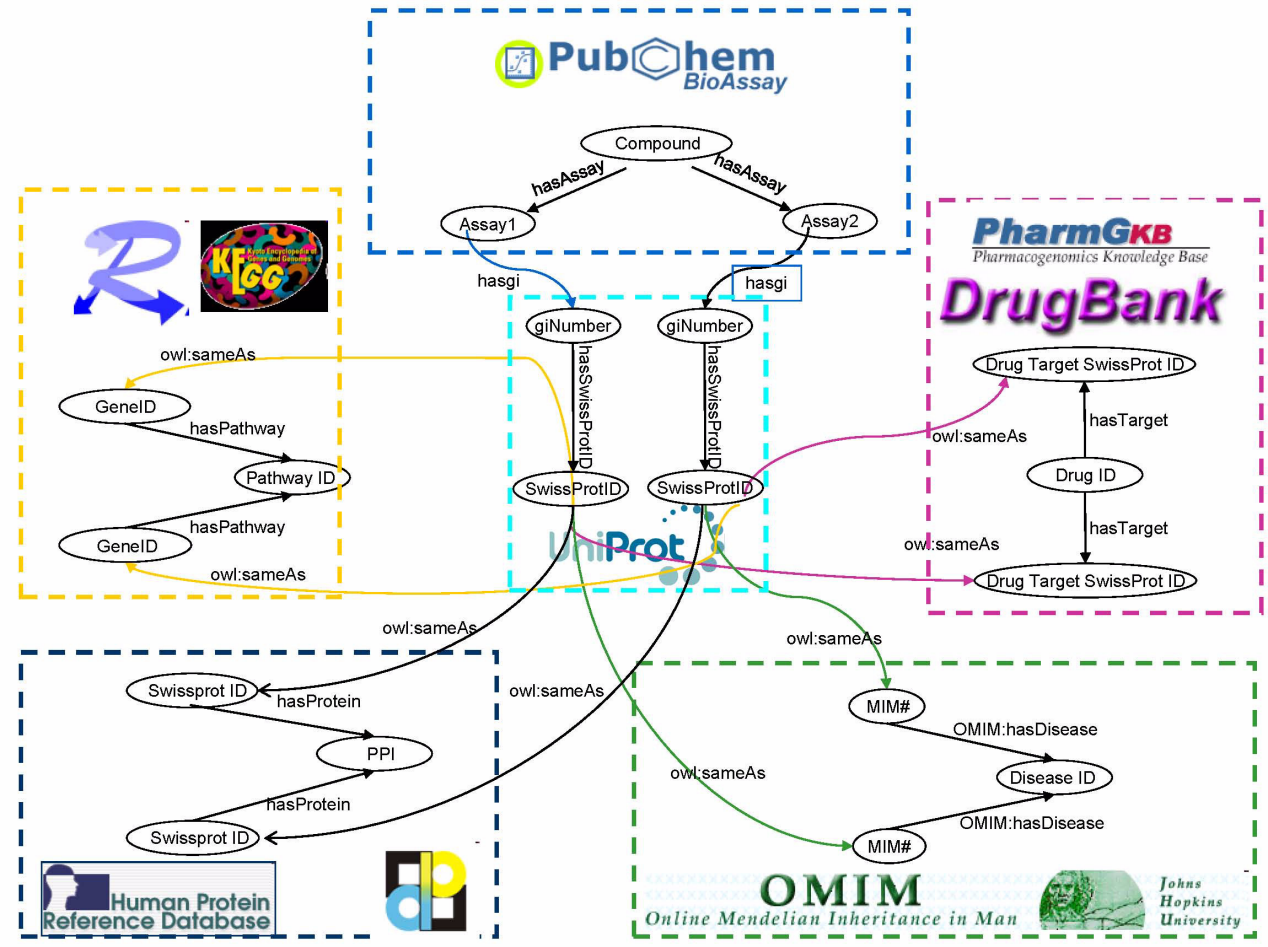
**Class links for polypharmacology**. Includes the classes: Bioassay, Drug Target, Pathway, Protein-Protein Interaction, and Disease. Some classes include more than one data source. Two nodes in different classes are linked through two paths. For instance, drug *X *is linked to compound *Y *if targets *A *and *B *of drug *X *are linked to assays *A *and *B *of compound *Y *via UNIPROT ID.

**Figure 5:**
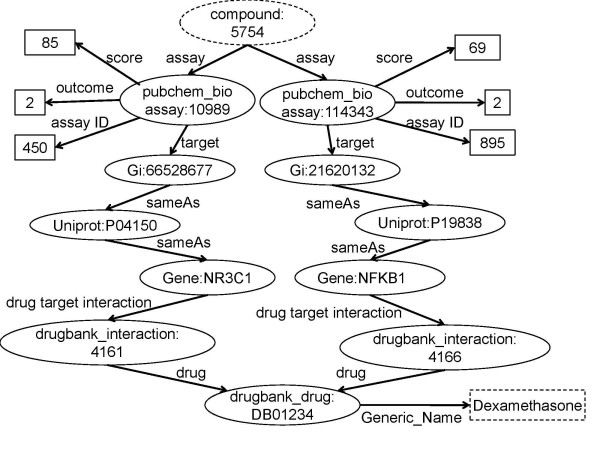
**Graphical representation of the SPARQL query for Case Study 1**. PubChem compounds (e.g. CID 5754) are identified that are active in bioassays that are associated with protein targets, which are associated with genes (via UNIPROT), which are identified as those with which Dexamethasone interacts (via DrugBank). The resultant compounds are thus those that have a similar activity profile to Dexamethasone.

The query starts with retrieving the active compounds, followed by the identification of targets, which are then linked to drugs in DrugBank (Figure 4). In PubChem BioAssay, outcome represents the binary result (1 is inactive, 2 is active) and the normalized score measures the activity (0-100). We select the compounds with activity score greater than 50. In addition, since it is expected that retrieved compounds are drug-like, the function ruleofFive is used to filter only those compounds that pass four drug-likeness rules. One path is then created if the compound is able to link to the input drug (i.e., Dexamethasone) by sharing one common target, however, as Figure 4 shows, only compound that has at least two paths reaching the input drug shows polypharmacology, thus we group the paths, and select the compound with the number of link paths greater than 2 as the output. This query process is illustrated in Figure 5.

Nine of retrieved active compounds are active against at least two of the same protein targets, all of which are drug-like (in terms of Lipinski's Rule of Five). These compounds make sense from a medicinal chemistry perspective. For example, dexamethasone and one result tocris-1126 (CID: 6603742) have similar activities in NFKB1 and NR3C1, because they only have slight difference in stereochemistry. The activity of dexamethasone is also similar to that of another search hit, hydrocortisone (CID: 5754), where the addition of the methyl and fluorine to hydrocortisone has no effect on the activity but improves its drug-likeness as measured by the rule-of-five. In our generalized mapping process, we found 55 significant proteins in DrugBank that are studied in PubChem BioAssay. 27 drugs have corresponding active compounds showing polypharmacology.

### Case study 2: Linking KEGG/Reactome Pathways and PubChem to identify potential multiple pathway inhibitors for MAPK

**Figure 6:**
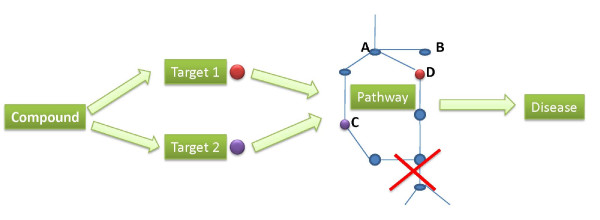
**Illustration of polypharmacology in pathways**. The compound is active against two proteins that are located in the two branches of the pathway that is associated with one disease. Targeting either node C or node D is not able to block the whole pathway.

Traditional drug discovery approaches focus on identifying a potential target in a disease-related biological pathway, and then finding a drug molecule to interact with this target. However, divergent and redundant pathways in humans often enable a system to keep functioning if one pathway is blocked as there is an alternative pathway to compensate [37]. This can get quite complex, as illustrated in Figure 6, where it is inappropriate to inhibit the upstream node A, which has a downstream node B that performs other biological functions. Therefore, in order to block the whole pathway, the drug has to inhibit targets C and D, which are located in the separated branches. If the compound in PubChem is active against C and D, it might be of interest to further investigate as it has efficacy toward the disease raised from the dysfunction of the pathway. We can therefore begin to identify such compounds with the question: *find all the compounds in PubChem that are active towards at least two targets that are in a given pathway*. We can formalize this into a Chem2Bio2RDF query firstly by generating a rule linking compounds with pathways via UniProt. This rule can be illustrated as: compound *x *is targeting protein *y*, and protein *y *belongs to pathway *z*, thus we reason that compound *x *is related to pathway *z*. We can then implement this rule in a SPARQL query (the rule and the query are supplied on the chem2bio2rdf.org website).

**Figure 7:**
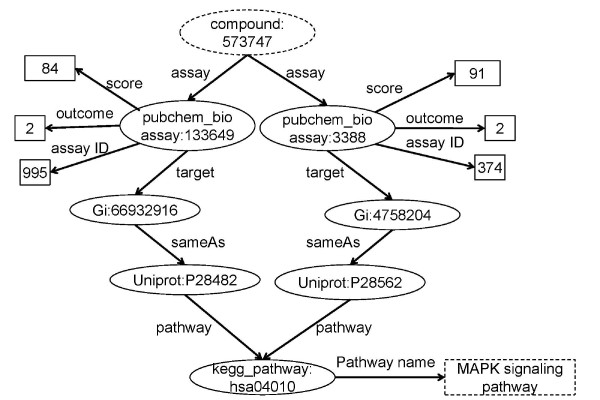
**Graphical representation of the SPARQL query for Case Study 2**. PubChem compounds (e.g. CID 573747) are identified that are active in bioassays that are associated with protein targets, which are associated with genes (via UNIPROT) which are identified as being part of the MAPK signalling pathway (via KEGG). We thus identify compounds which have multiple paths, and thus which interact with multiple targets in this protein.

The rule generates triples consisting of compound and pathway, which are further refined by its activity (outcome is 2) and pathway name (including MAPK signalling pathway). Finally, like the linking in case 1, the results are grouped and only compounds that are multiple pathway inhibitors are selected. This is illustrated in Figure 7.

The MAPK signalling pathway plays important roles in coordinating cell proliferation, differentiation and death. The inhibitors of proteins involved in the pathway are widely studied, but the robustness of this pathway leads to drug resistance. Cisplatin, for example, is used to treat ovarian cancer but the development of resistant cell population limits its efficiency in long-term trials. It has been suggested that targeting the ERK-MKP-1 system could destroy this pathway and further overcome Cisplatin resistance in human ovarian cancer treatment [38]. One compound (CID: 573747) was found in the retrieved results that has never been reported in the literature, but which can apparently inhibit both ERK2 and MKP-1. We might consider this a candidate to provide a new direction for the design of inhibitors of both ERK and MKP-1 to reduce Cisplatin resistance. After iterating all the known pathways, we hit 36 pathways, in which at least two proteins are inhibited simultaneously by at least one compound in PubChem.

### Case study 3: Linking KEGG and DrugBank to associate pathways with drug hepatotoxicity

Adverse drug reactions are of serious consequence and are often the subject of rigorous investigation in pharmaceutical R&D processes. Here, we apply Chem2Bio2RDF to study the most significant pathways that are associated with a given adverse drug reaction. The association between side effect and pathway is made using the pathways' gene components that are targets of drugs with known side effects. More specifically, we consider a gene is related to a certain side effect if and only if at least two drugs targeting this gene have reported the same side effect. Further, if there exists a pathway that contains more than 2 gene targets that are associated with that side effect, an associative relationship between the pathway and side effect can be drawn. Clearly, the more these associative paths can be discovered, the stronger the evidence of such pathway-adverse drug effect association it becomes.

In this study, we examined hepatotoxicity (liver toxicity) as the side effect. Drug induced liver injury is a major cause of safety-related drug withdrawal (e.g., Ticrynafen, Benoxaprofen, Bromfenac, Troglitazone, Nefazodone) both before and after a drug goes to market, and thus identifying pathways in the body that might be associated with liver function and toxicity is important. Here we define drugs associated with hepatotoxicity as those with side effect terms that include *necrosis*, *hepatitis *and *hepatomegaly*.

We posed the specific question: *find the top 5 pathways in the KEGG pathway dataset that contain at least two efficient targets that have drugs that are associated with hepatotoxicity*. A gene target is considered as efficient if the gene is targeted by at least two drugs that cause the given side effect. This question can be formed into a SPARQL query which is available on the chem2bio2rdf.org website.

**Figure 8:**
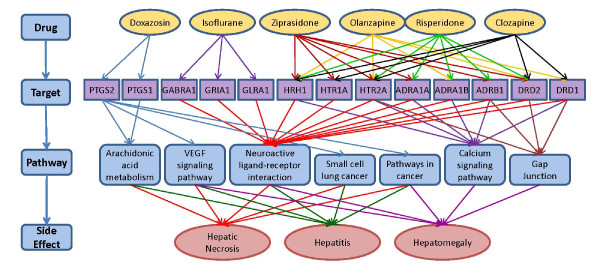
**Associating pathways with hepatotoxic effects**. The drugs that are associated with hepatotoxicity-related side effects are associated with their targets using DrugBank. The targets are associated with pathways using KEGG to establish association chains between pathways and side-effects.

The graph linking these terms, pathways, targets and drugs is shown in Figure 8, which includes the top 5 pathways identified from the search. They share the top 5 pathways: Arachidonic acid metabolism, VEGF signalling pathway, Neuroactive ligand-receptor interaction, small cell lung cancer, and pathways in cancer. The mechanism for hepatomegaly is slightly different. The top 5 pathways of hepatomegaly contain the calcium signalling and gap junction pathway, which are not involved in the hepatic necrosis and hepatitis. Literature review [39] shows that those pathways are highly correlated with liver injury. For instance, the increase concentration of calcium in the calcium signalling pathway will cause the damage of hepatic cell. The targets we discovered are also known as the major targets for liver injury based on literature reviews [40].

## Discussion

The difficulties of polypharmacology are to explore the combination of targets and then to identify active compounds against the sets of targets. Linking between chemical, biological, systems, and phenotype data is demonstrated to be a promising way to address the problems. For example, linking between bioassay data and market drug data enables to explore the compounds similar to drugs that already show polypharmacology. Quinacrine, which has been used as an anthelmintic and in the treatment of giardiasis and malignant effusions, shows polypharmacology. One compound Loxapine (CID: 71399) is found to show similar polypharmacology with quinacrine. Loxapine is active in both BioAssay 859 and BioAssay 377, whose targets are CHRM1 and ABCB1 respectively. As Loxapine tends to be hydrophobic, medicinal chemists would not be surprised that it is active in BioAssay 377, which identifies substrates (or inhibitors) for multidrug resistance transporter. It is also reported that Loxapine might get metabolized to Amoxapine that is a considerably weak antagonist in BioAssay 859 [41]. Other than Loxapine, many identified compounds such as Oxybutynin were proved to show polypharmacology by literature reviews.

By linking bioassay data to pathways, we can identify the compounds that inhibit at least two of proteins in a pathway, leading to the pathway dysfunction. For example, compound CID 6419769 could interact with proteins HSD11B1 and AKR1C4, which are in the different branches of C21-Steroid hormone metabolism pathways. The blocking of the pathway might be able to partially explain why CID 6419769 has side effects [42]. In protein-protein interaction networks, two proteins are connected if both physically interact. In terms of polypharmacology, the deletion of one protein does not affect the whole network, but if two connected nodes with high degree were deleted, the network would be disturbed. For example, by linking bioassay to PPI, we found that two compound (CID: 460747 and CID: 9549688) are active against two high degree proteins (PLK1 and TP53) which are associated with cancer.

We note that there are parallel contributions from different data sources and vendors (for example, KEGG and Reactome both provide pathway data). We think that an important part if this work is not just the integration of heterogeneous data, but also the integration of sources covering homogenous kinds of data. Tables 2, 3, 4 show the percentage contribution of unique information for homogeneous sources for protein-protein interaction (PPI), pathway, and chemogenomics data respectively. For PPI, HPRD and DIP have 35645 and 32976 unique protein pairs respectively, and the total number of unique pairs in two datasets is 67769. Each dataset contributes almost half of the pairs, and both share very little number of common pairs. The PPI network would not be complete if either dataset were ignored. Pathway is more complicated than PPI, since each organization could have its own definition of pathway, which makes the whole integration very difficult. For example, a pathway in Reactome is usually composed by a small number of proteins, although the total number of pathways is more than KEGG, the proteins involved in Reactome are far less than KEGG. We are not able to judge which one is better, thus we have to consider all pathway datasets together. For the chemogenomics data, a chemical protein interaction is recorded as one entry, and all the unique interactions were derived from 6 datasets. We did not consider another two chemogenomics data sets (KEGG Ligand and PharmGKB), as KEGG Ligand includes only metabolic molecules rather than chemicals designed for drug discovery and many drugs in PharmGKB only provide names from which the chemical identifier is not able to be linked to compound. Each dataset only contributes a small portion of interactions so that it is not able to represent all chemogenomics data. PubChem BioAssay uses high throughput screening which allows testing thousands of compounds per experiment, thus yielding a large number of chemical protein interactions, but the number of targets studied in PubChem is small compared CTD. The benefit of integration has ramifications for linking too. For example, if we take an example in linking *chemical *to *pathway *via chemogenomics data, chemical has 6 directions (6 chemogenomics datasets) to associate with a gene that is mapped to multiple pathways in either KEGG or Reactome. We randomly picked up 100 drugs from SIDER and linked the drug to pathway through 6 chemogenomics datasets. In another four experiments, we only selected one dataset for one domain instead of using all datasets. If only CTD is selected for chemogenomics data and only KEGG is selected for pathway, the number of paths linking from the 100 drugs to pathways and the number of pathways we found are 6,863 and 178 respectively, compared to 12,240 and 350 when all chemogenomics and pathway data sources were selected (Table 5).

**Table 2:** PPI data source distribution.

Data source	# of records	percentage
HPRD	35645	52.6%

DIP	32976	48.7%

ALL	67769	

**Table 3:** Pathway data source distribution.

Data Source	Protein records		Pathway records	
KEGG	8172	81.0%	192	34.8%

Reactome	4397	43.6%	360	65.2%

ALL	10091		552	

**Table 4:** Chemogenomics data source distribution

Data source	# of records	percentage
BindingDB	36839	12.1%

CTD	95786	31.5%

DrugBank	10381	3.4%

Matador	15843	5.2%

PubChem	146088	48.1%

QSAR	2148	0.7%

ALL	303773	

**Table 5:** Results of linking sample drugs to pathways.

Dataset used	paths	genes	pathways
CTD, KEGG	6863	763	178

CTD, Reactome	1157	547	146

PubChem, KEGG	522	33	86

PubChem, Reactome	97	24	18

ALL	12240	1181	350

## Conclusions

We have created a new systems chemical biology resource called Chem2Bio2RDF that integrates small molecule, target, gene, pathway and drug information and permits cross-source linking with LODD and Bio2RDF. We have demonstrated the utility of this approach in specific examples of polypharmacology, multiple pathway inhibition, and adverse drug reaction - pathway mapping. We also demonstrated the usefulness of extending the SPARQL query language with cheminformatics and bioinformatics functionality, and have discussed the importance of integrating not just heterogeneous data but data sources which cover the same kinds of data.

We propose three further developments of this work. First, we hope to include more resources and datasets into Chem2Bio2RDF as they become available. Second, we see a variety of applications of using large-scale identification and ranking of paths of interest between data sources, and we are working on developing methods for this. Third, we are linking Chem2Bio2RDF with a variety of network and data visualization tools.

## Authors' contributions

BC carried out the majority of the implementation of Chem2Bio2RDF, supervised by YD and DJW. BC, HW and QZ created the data sources. XD worked on the architecture. DJ developed the SPARQL extensions. HW worked on the case 3 study. DJW assembled this paper from a previous white paper written by BC, YD and DJW. All contributed to the intellectual evolution of this project. All authors have read and approved the final manuscript.
